# Dietary free L-glutamate contributes to maintaining a low sodium intake among Vietnamese

**DOI:** 10.3389/fnut.2024.1352832

**Published:** 2024-07-17

**Authors:** Vu Thi Thu Hien, Le Danh Tuyen, Andrea Wakita, Saiko Shikanai, Le Thi Hang, Nguyen Thi Diep Anh, Nguyen Thi Anh Nguyet, Tamami Iwamoto, Hideki Matsumoto, Hisayuki Uneyama, Nguyen Vu Son, Nguyen Nhat Linh, Shigeru Yamamoto

**Affiliations:** ^1^Vietnam National Institute of Nutrition (NIN), Hanoi, Vietnam; ^2^Ajinomoto Co., Inc., Institute for Innovation, Umami Group, Kawasaki, Japan; ^3^Asian Nutrition and Food Culture Research Center, Jumonji University, Niiza, Japan; ^4^International Nutrition, Department of Food and Nutritional Sciences, Jumonji University, Niiza, Japan; ^5^Hanoi Medical University, Hanoi, Vietnam; ^6^University of Science and Technology of Hanoi, Hanoi, Vietnam

**Keywords:** free L-glutamate, umami, sodium intake, cross-over, dietary, maintain

## Abstract

**Background:**

The World Health Organization (WHO) and the Food and Agriculture Organization (FAO) recommend the consumption of less than 2,000 mg of sodium/day to reduce blood pressure and the risk of conditions such as cardiovascular disease and coronary heart disease. The sodium intake among Vietnamese was reported to be 7,200 mg/d or more. Free L-glutamate enhances flavor when it is added to food and improves the taste of sodium-reduced foods.

**Objective:**

This study aims to investigate whether the intake of free L-glutamate-rich seasonings contributes to maintaining a low sodium intake in a cross-over study.

**Methods:**

From a total of 145 subjects, 42 participants were screened for participation in the cross-over design study. Subjects were randomly allocated to the Low free L-glutamate group (Low free L-Gl) and the Normal free L-glutamate group (Normal free L-Gl). Both received a direct educational guideline to reduce sodium intake. The Low free L-Gl group started with a restriction in the variety of free L-glutamate-rich seasonings, and the Normal free L-Gl group had no restriction in the variety of seasonings. Blood pressure was measured at week 0 (baseline), week 2, week 4, and week 6, while body weight, height, urine sodium and potassium excretion, chromogranin-A (CgA pmol/mg protein) from saliva, and free L-glutamate from food were measured at week 0, week 3, and week 6.

**Results:**

In Low free L-Gl, the amount of free L-glutamate in food decreased significantly from baseline to week 6 (*p* < 0.00), while it did not change in the Normal free L-Gl (*p* > 0.05). However, the reduction of sodium excretion at week 6 was 22% in Low free L-Gl (5,875 mg/d vs. 4,603 mg/d, *p* < 0.01) and 46% in Normal free L-Gl (6,107 mg/d vs. 3,277 mg/d, *p* < 0.00), both lower than the baseline. CgA (pmol/mg protein) did not show any difference between the two groups.

**Conclusion:**

The group with Normal free L-Gl intake showed a 46% reduction in sodium excretion by week 6 compared to the baseline. This suggests that the consumption of L-glutamate-rich seasonings when complemented with direct educational guidelines, can contribute to maintaining a low sodium intake.

## 1 Introduction

WHO and FAO recommend the consumption of less than 2,000 mg of sodium/day. This expert recommendation emphasizes that the highest number of diet-related deaths are associated with excessive sodium intake, which increases blood pressure levels and thus increases the risk of cardiovascular disease (1). Studies have found a positive correlation between sodium intake and blood pressure, and reducing sodium intake is considered a non-pharmacological approach to the prevention and treatment of hypertension (2–4). Public health and nutrition authorities around the world have set dietary goals for sodium intake and have been attempting to implement a wide variety of sodium reduction initiatives, such as labeling high-sodium products in a way that clearly informs consumers about the sodium content of a product (1, 5). However, to date, no country or organization has achieved its goals (6).

In Vietnam, the prevalence of hypertension was 23.9% among men and 13.7% among women aged 25–64 years (7). The prevalence was higher among elderly people; it was present in 43.8% of men and 35.5% of women aged 60 to 69 years living in the urban area of Ho Chi Minh City (8). In central Vietnam, it was reported that the prevalence of hypertension was 51% in men and 40% in women aged between 40 and 69 years (9). The sodium intake in Vietnam was reported to be 7,200–8,800 mg/d; thus, Vietnam is in urgent need of intervention to control hypertension and related chronic diseases (10). However, for many people who attempt a sodium-reduced diet, the decrease in taste could increase their internal stress and cause them to abandon the diet before adapting to the taste perception. Iwamoto et al. reported that in a sodium-reduced diet without the addition of umami seasoning (free L-glutamate), the salivary sympathetic system marker concentration, chromogranin-A (CgA pmol/mg protein), which is represented as a percentage of change from baseline, was higher in the sodium-reduced diet without the addition of umami seasoning than the sodium-reduced diet with the addition of umami seasoning (11).

Free L-glutamate has flavor-enhancing properties by which, it enhances the perception of continuity of flavor when it is added to food (12). The free L-glutamate is present in high concentration in foods such as cheese, mushrooms, tomatoes (13), and fermented seasonings (14), and it has been reported that umami seasoning improves the taste of sodium-reduced foods (15–17). A physiological study to explain this mechanism was conducted by Onuma et al. It measured brain activities using functional near-infrared spectroscopy (fNIRS) and showed that the added umami seasoning in sodium chloride solution enhanced the hemodynamic response in temporal brain regions but did not alter the responses of the parotid salivary glands. These results suggested that the perceived enhancement of the saltiness effect occurred in the brain region, which is involved in the central gustatory processing (18). At present, the relationship between free L-glutamate and blood pressure has been studied previously (19); however, there are no data on total free L-glutamate intake and its effect on the reduction of sodium intake in a daily diet and on the impact on stress when dietary sodium is reduced in free-living subjects with prehypertension and hypertension. Thus, this study was conducted to investigate whether the intake of free L-glutamate-rich seasonings can contribute to maintaining a low sodium intake in a cross-over study design in which subjects received a direct educational guideline for reducing sodium.

## 2 Materials and methods

The study was approved by the Research and Ethical Committee of the Vietnamese National Institute of Nutrition (NIN). Written informed consent was obtained from all participants before conducting the survey. This study was registered at the UMIN-CTR Clinical Trial (UMIN000042814).

### 2.1 Baseline survey to screen and select participants for the cross-over study

A multistage sampling method was used to select participants. The capital city, Hanoi, was chosen as the site for conducting the study from September 2011 to January 2012. Among the suburban areas of Hanoi, Long Bien district (a new district of Hanoi) was selected. Then, two wards were chosen randomly from the list of wards in the district. Furthermore, lists of all households in the selected wards were established, and family codes were created. From this list, households in which all family members usually took their meals at home and had at least one member aged ≥45 years were randomly picked among family codes. In each household chosen, all family members aged ≥20 years were invited to be examined for clinical symptoms and answer a questionnaire on general information, such as age, education level, health history, physical situation, and dietary intake to screen and select subjects for the cross-over study. Individuals were excluded if they had any of the following factors: malformation; chronic or acute disease; pregnant and lactating women; on a special diet for weight loss, weight gain, vegetarianism, salt reduction, diabetes mellitus, or other reasons. The survey was conducted by trained researchers and doctors who are NIN staff. In interviews and examinations, the doctors employed a specially designed questionnaire that included questions related to weight, height, blood pressure, demographics, occupation, and physical activities. The medical history of each participant was also requested. Finally, 145 subjects were screened participants for the cross-over study.

### 2.2 The cross-over study

#### 2.2.1 Design of the study

**Figure 1:**
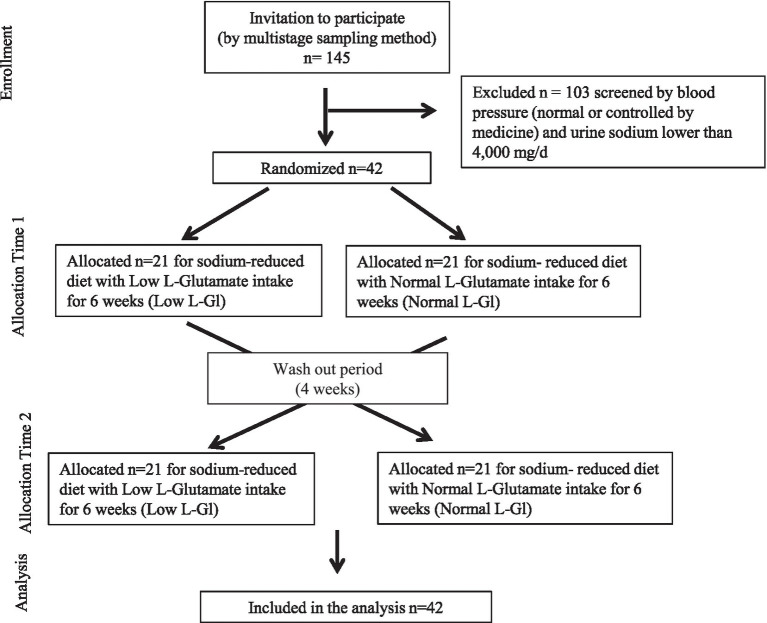
CONSORT diagram of the study design.

Out of the 145 subjects, those who were identified with prehypertension or hypertension (systolic blood pressure 120–139 mmHg or diastolic blood pressure 80–90 mmHg) (20) and had urinary sodium of ≥4,000 mg/d were selected for the study (*n* = 42). Subjects were randomly allocated to a group that would have a low content of free L-glutamate seasonings (Low free L-Gl) or to a group that would have a normal amount of free L-glutamate (Normal free L-Gl) to participate in a cross-over design for 16 weeks (Figure 1). Both groups received the same direct educational guideline to take less than 2,000 mg/d of sodium from salt, seasonings, and processed food and also raised awareness of the health consequences of having high blood pressure, the importance of a reduced-sodium diet in controlling high blood pressure, and a list of foods rich in sodium. The Low free L-Gl group started using only soy sauce as a free L-glutamate source. Other seasonings without free L-glutamate were allowed, such as salt, oil, and chili. The Normal free L-Gl group started using an array of seasonings without any restrictions on variety.

#### 2.2.2 Assessment of variables

Data were collected by trained researchers and doctors at the participants’ homes.

Blood pressure measurements were measured at baseline (week 0, “W0”), week 2 (“W2”), week 4 (“W4”), and week 6 of intervention (“W6”) using random-zero sphygmomanometer devices. Blood pressures were taken at least 1 h after eating, with participants in a seated position and using the right arm. Two measurements were taken at each visit. The first measure was taken after a 5-min period of quiet sitting, and the second was taken 30 s after the first. Smoking, drinking caffeinated beverages, and vigorous exercise were not allowed 30 min before the measurement of blood pressure.

Assessment of individual dietary intake was made using the 24 h recall method and the weighting method on 3 consecutive days (including weekends). The staff went to participants’ homes on the 3 days to collect samples and to freeze them as soon as possible during W0, week 3 (“W3”), and W6. Free L-glutamate intake was measured by trained NIN staff using high-performance liquid chromatography (HLPC).

Sodium and potassium intake was calculated from 24 h urine excretion for 3 consecutive days during W0, W3, and W6. The urine was collected by subjects with a special device that was easy to use (Akita Sumitomo Bakelite Co., Ltd., Japan). The urine samples were gathered every day by NIN staff, transported to the NIN laboratory, and stored at −20 °C until analysis. Sodium concentrations in urine were analyzed at NIN.

### 2.3 The measurement of free L-glutamate in foods by HPLC

The free L-glutamate in foods was measured by HPLC (Agilent, United States). The HPLC method was validated for accuracy, precision, linearity range, limit of detection (LOD), and limit of quantification (LOQ) according to ICH guidelines (21).

The procedures to collect the foods from subjects’ meals for analysis of total free L-glutamate were performed as follows: subjects were asked to prepare a double amount of the food they would consume and separate into two plates with the same amount of food in each plate. Subjects ate from one of the plates, and another one was stored in the refrigerator at the participants’ home. Subjects were asked to keep all the food they left on the plate until the NIN staff came to measure the residual amount and take the amount eaten from the sample dishes. A protocol was provided to the participants. The same process was conducted at W0, W3, and W6. This amount was analyzed to assess the free L-glutamate in food. To cover the cost of the extra meal (double meal), the NIN reimbursed the household for it. Staff in the laboratory of NIN measured the free L-glutamate from the meal.

### 2.4 Stress marker from saliva (chromogranin A pmol/mg protein)

Saliva was collected at W0, W3, and the last day of the intervention period (W6). Subjects need to wake up and take the salivary sample at the same time. A saliva sample had to be taken within an hour of waking up using a saliva collector. Subjects chewed the cotton for 3 min and then deposited it in the sample tube (Salivette, Sarstedt, Germany). The concentration of saliva chromogranin A (pmol/mg protein), CgA (pmol/mg protein), was determined by Yanaihara Institute Inc., Japan.

### 2.5 Statistical analyses

All analyses were performed using the IBM^®^ Statistical Package for Social Science (version 27.0.1.0; SPSS). All data were expressed as mean and standard error (SE). Shapiro–Wilk test was used to test the normality of distribution. Since data did not have a normal distribution, differences between means were evaluated using the non-parametric Wilcoxon test when the Low free L-Gl group was compared with the Normal free L-Gl group; and W0 was compared with W3 and W6 for free L-glutamate intake, urine sodium and potassium excretion, the ratio of urinary sodium/potassium excretion, and Cg A; and W0 was compared with W2, W4, and W6 for blood pressure. A *p*-value of <0.05 was considered significant.

## 3 Results

Out of the 145 Vietnamese selected as possible participants, after screening for subjects to participate in the cross-over study according to their sodium intake level (higher than 4,000 mg/d) and the presence of at least prehypertension, a total of 42 subjects were selected for the cross-over study.

### 3.1 The characteristics of the subjects at baseline for the cross-over study

The characteristics of the screened subjects (*n* = 42) and the group with Low and Normal free L-GIu intake are described in Tables 1, 2.

**Table 1:** Characteristics of the population at baseline (*n* = 42).

Variables		All subjects (*n* = 42)		Low free L-Gl (*n* = 21)		Normal free L-Gl (*n* = 21)		*p-*value Low vs. Normal free L-Gl
		Mean	SE	Mean	SE	Mean	SE	
Age	y-old	61.8	1.0	64.0	0.9	59.5	1.7	0.04
BMI	kg/m^2^	23.0	0.4	23.2	0.7	22.7	0.5	0.79
Systolic blood pressure	mmHg	130.2	1.1	130.5	1.6	129.9	1.5	0.68
Diastolic blood pressure	mmHg	80.7	0.8	81.1	1.0	80.3	1.2	0.86
CgA (Cg A/ Protein)	pmol/mL	13.6	1.1	10.7	1.2	16.4	1.6	0.01
Free-glutamate intake from food	mg/d	1,840	252	1,572	353	2,108	358	0.07
24 h urine sodium excretion	mg/d	7,153	288	7,335	480	6,971	326	0.79
24 h urine potassium excretion	mg/d	2,401	236	2,567	394	2,234	264	0.40

Mean ± standard error (SE) of age, BMI, blood pressure, saliva CgA, free L-glutamate, and urine sodium excretion during the baseline. Statistical analyses were performed using the Wilcoxon test to compare the baseline means of those who were allocated to the Low free L-Gl group (Low free L-glutamate intake) with the Normal free L-Gl group (Normal free L-glutamate intake).

**Table 2:** Demographic characteristics and physical activities of subjects at baseline (*n* = 42).

Variables			All subjects (*n* = 42)	Low free L-Gl (*n* = 21)	Normal free L-Gl (*n* = 21)
Gender	Male	%	54.8	57.1	52.4
	Female	%	45.2	42.9	47.6
Occupation	Officer	%	14.3	14.3	14.3
	Free worker	%	4.8	0	9.5
	Housewife	%	2.4	0	4.8
	Retired	%	50	66.7	33.3
	Other	%	28.6	19	38.1
Walking or cycling for at least 10 min per day	Yes	%	73.8	81	66.7
	No	%	26.2	19	33.3

Percentage of subjects for each category.

For the total subjects (*n* = 42), the mean age was 61.8 ± 1.0 years (Table 1), BMI was 23 ± 0.4 kg/m^2^, systolic blood pressure was 130.2 ± 1.1 mmHg, diastolic blood pressure was 80.7 ± 0.8 mmHg, and CgA (pmol/mg protein) was 13.6 ± 1.1 pmoL/mL. The mean free L-glutamate intake at baseline was 1,840 mg/d ± 252, and the mean urinary sodium and potassium excretion were 7,153 ± 480 mg/d and 2,401 ± 236 mg/d, respectively (Table 1).

Demographics, occupations, physical activities, and lifestyle characteristics showed that 54.8% of subjects were men, 50% were retired, and 73% of subjects performed walking or cycling for at least 10 min per day (Table 2).

After randomly allocating each subject to the Low free L-Gl and Normal free L-Gl groups, the demographics, occupations, physical activities, lifestyle characteristics, BMI, free L-glutamate intake, urine sodium and potassium excretion, and blood pressure did not show a statistical difference between the Low free L-Gl and Normal free L-Gl groups (Tables 1, 2). On the other hand, age and CgA were significantly different between the two groups (*p* = 0.035 and *p* = 0.011, respectively).

### 3.2 Intervention phase

**Figure 2:**
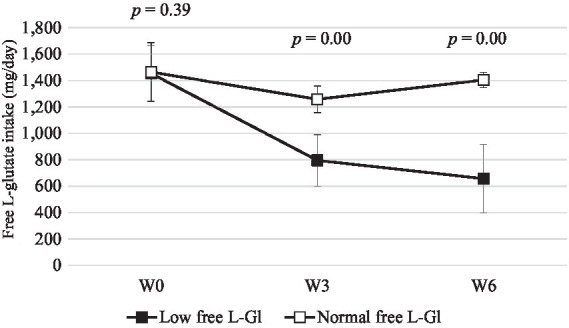
Free L-Glutamate intake (mg/day) (mean ± standard error) at W0: week 0 (baseline), W3: 3 weeks of the intervention phase, and W6: 6 weeks of the intervention phase (*n* = 42). Statistical analyses were performed using the Wilcoxon test to compare the mean of Low free L-Gl (■) with Normal free L-Gl (□). The *p*-value of <0.05 was considered statistically significant.

**Figure 3:**
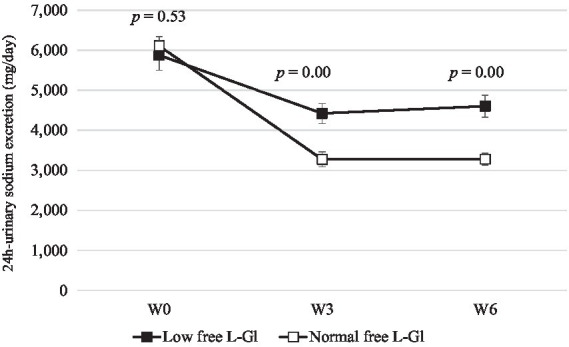
24 h urine sodium excretion (mg/day) (mean ± standard error) at W0: week 0 (baseline), W3: 3 weeks of the intervention phase, and W6: 6 weeks of the intervention phase (*n* = 42). Statistical analyses were performed using the Wilcoxon test to compare the mean of Low free L-Gl (■) with Normal free L-Gl (□). The *p*-value of <0.05 was considered statistically significant.

**Figure 4:**
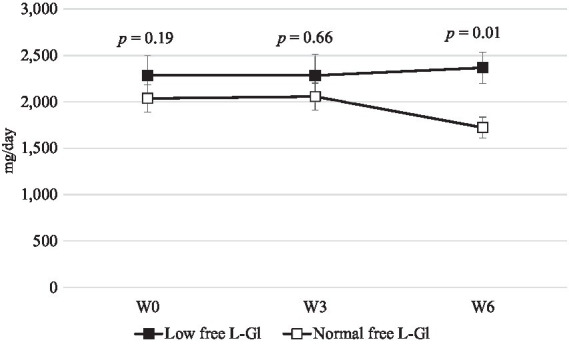
24 h urine potassium excretion (mg/day) (mean ± standard error) at W0: week 0 (baseline), W3: 3 weeks of the intervention phase, and W6: 6 weeks of the intervention phase (*n* = 42). Statistical analyses were performed using the Wilcoxon test to compare the mean of Low free L-Gl (■) with Normal free L-Gl (□). The *p*-value of <0.05 was considered statistically significant.

Figure 2 shows that free L-glutamate intake was lower in the Low free L-Gl group during the intervention phase. In addition, the intake of free L-glutamate was significantly lower at W3 and W6 compared to the baseline (*p* = 0.00) in the Low free L-Gl group, while the Normal free L-Gl group did not show any statistical difference from the baseline (Table 3). When urine sodium and potassium excretion were compared between the two groups (Figures 3, 4), the sodium was significantly lower at W3 (*p* = 0.00) and W6 (*p* = 0.00) and the potassium at W6 (*p* = 0.01) in the Normal free L-Gl group. In addition, the urine sodium excretion decreased significantly when comparing W0 with W3 and W6 in both the Low free L-Gl and Normal free L-Gl groups (Table 3). On the other hand, the urine potassium excretion did not show a statistically significant change during the intervention phase in the Low free L-Gl group (W0, W3, and W6), but in the Normal free L-Gl group, the urine potassium excretion at W6 was significantly lower than at W0 (Table 3). The ratio of urinary sodium/potassium excretion significantly decreased from W0 to W3 and from W0 to W6 in both groups.

**Table 3:** Free L-glutamate, 24 h urinary sodium and potassium excretion, blood pressure, and saliva CgA during the intervention phase in the Low and Normal free L-glutamate intake groups (*n* = 42).

Variable			Low free L-Gl			Normal free L-Gl		
			Mean	SE	*p*-value	Mean	SE	*p-*value
Free L-Glutamate intake from food	mg/d	W0	1,453	222		1,464	211	
		W3	795	100	0.00	1,258	196	0.07
		W6	657	59	0.00	1,404	259	0.56
24 h urine sodium excretion	mg/d	W0	5,875	372		6,107	237	
		W3	4,422	248	0.00	3,273	185	0.00
		W6	4,603	275	0.01	3,277	149	0.00
24 h urine potassium excretion	mg/d	W0	2,284	214		2,036	147	
		W3	2,284	230	0.60	2,056	146	0.68
		W6	2,366	169	0.26	1,722	113	0.03
24 h urine sodium/potassium excretion	mg/mg	W0	3.0	0.2		3.5	0.2	
		W3	2.3	0.2	0.01	2.0	0.3	0.00
		W6	2.1	0.1	0.01	2.1	0.1	0.00
Systolic blood pressure	mmHg	W0	130.3	1.3		129.2	1.1	
		W2	129.8	1.3	0.77	128.1	1.3	0.43
		W4	130.0	1.2	0.74	128.0	1.1	0.24
		W6	130.1	1.6	0.79	126.9	1.1	0.03
Diastolic blood pressure	mmHg	W0	80.4	0.8		79.2	0.8	
		W2	78.5	0.8	0.04	78.5	1.0	0.33
		W4	80.1	0.9	0.59	79.4	0.7	0.69
		W6	80.8	1.1	0.84	78.9	0.8	0.73
CgA (Cg A/Protein)	pmol/ml	W0	14.6	1.3		15.5	1.8	
		W3	14.4	1.1	0.75	16.1	1.7	0.97
		W6	20.1	3.9	0.50	15.3	1.6	0.68

Data are expressed in mean ± standard error (SE). W0 was compared with W3, and with W6 for free L-glutamate intake, 24 h sodium and potassium excretion, 24 h urine sodium/potassium excretion and CgA (Cg A/Protein), and W0 was compared with W2, W4 and with W6 for blood pressure using Wilcoxon test. The *p*-value of < 0.05 was considered statistically significant. W0: week 0 (baseline), W2: second week, W3: third week, W4: fourth week, W6: sixth week of intervention phase.

**Figure 5:**
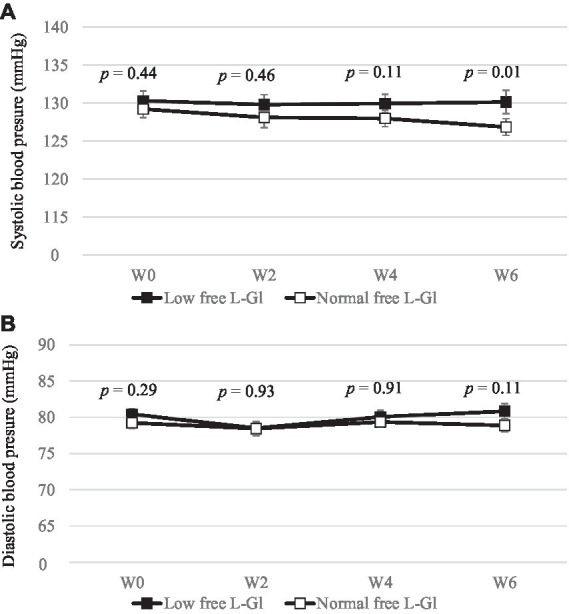
Systolic **(A)** and diastolic **(B)** blood pressure (mmHg) (mean ± standard error) at W0: week 0 (baseline), W2: 2 weeks of intervention phase, W4: 4 weeks of intervention phase and W6: 6 weeks of the intervention phase (*n* = 42). Statistical analyses were performed using Wilcoxon test to compare the mean of Low free L-Gl (■) with Normal free L-Gl (□). The *p*-value of < 0.05 was considered statistically significant.

**Figure 6:**
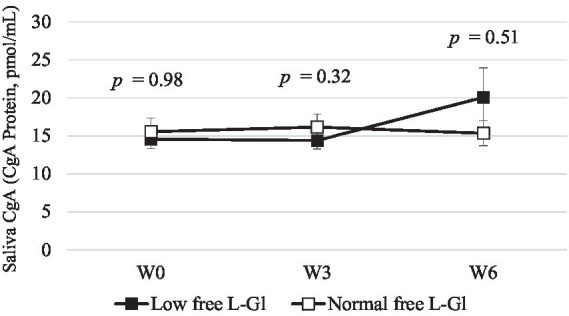
Saliva CgA (CgA Protein, pmol/mL) (mean ± standard error) at W0: week 0 (baseline), W3: 3 weeks of intervention phase and W6: 6 weeks of the intervention phase (*n* = 42). Statistical analyses were performed using Wilcoxon test to compare the mean of Low free L-Gl (■) with Normal free L-Gl (□). The *p*-value of < 0.05 was considered statistically significant.

The systolic blood pressure was significantly lower (*p* = 0.01) at W6 in the Normal free L-Gl group (130.1 ± 10.1 mm Hg) than in the Low free L-Gl group (126.9 ± 7.3 mm Hg) (Figure 5A), and the systolic blood pressure was significantly lower at W6 in the Normal free L-Gl group compared to the baseline (*p* = 0.03); however, the diastole blood pressure (Figure 5B and Table 3), and the stress marker (Figure 6 and Table 3) did not showed any significant difference.

## 4 Discussion

The screening of the 42 subjects for the cross-over study was conducted as shown in the consort of this study (Figure 1). Since age and CgA values were not criteria for inclusion, the statistical differences found at the baseline between the Low free L-Gl and Normal free L-Gl groups were not considered when allocating the subjects into one of the groups.

### 4.1 Free L-glutamate intake

Figure 2 shows that the free L-glutamate intake was statistically significantly lower in the Low free L-Gl group than in the Normal free L-Gl group. Free L-glutamate is an umami compound that can enhance the taste and flavor of low-sodium foods (15), making them more palatable without the need to add more sauces or salt. In Asian food culture, there are several seasonings rich in umami, such as fermented fish sauce, oyster sauce, and soy sauce, which are also high in sodium content. These seasonings are used for cooking or as dipping sauces. Vietnam, as an Asian country, has this food culture (22). Despite sodium restrictions during the intervention phase, subjects from the Normal free L-Gl group were permitted to use these seasonings. To respect the food culture, in the Low free L-GL group, soy sauce was allowed.

### 4.2 Urinary sodium and potassium excretion

Figure 3 shows clearly that the sodium intake in the Low free L-Gl group was higher than in the Normal free L-Gl group. The urine sodium levels of both groups decrease significantly from W0 to W3 and from W0 to W6. This result suggests that the sodium intake of participants decreased after receiving educational guidelines. However, comparing both groups, the Low free L-Gl group had higher urine sodium excretion at W3 (*p* = 0.00) and W6 (*p* = 0.00) than the Normal free L-Gl group, despite there being no difference at W0 (*p* = 0.53). In addition, in the Low free L-Gl group, at W6, a 22% reduction in sodium was observed from the baseline, while in the Normal free L-Gl group, it was as much as 46%. This suggests that the group using a wide variety of umami-rich seasonings was able to maintaining a lower sodium intake. In this study, participants had the freedom to cook and add their preferred seasonings, following the seasoning guidelines provided for each phase. This methodology differs from the study by Iwamoto et al. (11), in which all meals were provided during the intervention, and participants were not allowed to add their own seasonings. Therefore, the results of this study could suggest that participants can effectively control their seasoning use without needing all meals to be provided, as was the case in the study by Iwamoto et al. (11). Those who participated in this study received educational guidelines on reducing their sodium intake. Since the primary aim of this trial was to investigate the relationship between free L-glutamate intake and urine sodium excretion, the consumption of potassium-rich foods was not promoted as an additional dietary strategy to decrease cardiovascular disease risk (23). Despite the recommendation of WHO for consuming >3,510 mg/d of potassium (23) and maintaining a sodium/potassium ratio of ≤0.6 mg/mg, this study found a higher ratio. Nevertheless, this ratio significantly decreased during the intervention phase due to a reduced urine sodium excretion observed in both groups. Therefore, it would be advisable for health programs aiming to reduce cardiovascular disease risk not only to provide education on the restriction of sodium intake but also to include the promotion of potassium-rich food consumption.

### 4.3 Blood pressure

Systolic blood pressures decreased significantly from W0 (129.2 ± 1.1 mmHg) to W6 (126.9 ± 1.1 mmHg) in the Normal free L-Gl group (*p* = 0.03), while there was no significant difference in the Low free L-Gl group (*p* = 0.79). In addition, at W6, this indicator was higher in the Low free L-Gl group than in the Normal free L-Gl group (*p* = 0.01) (Figure 5A). These changes in systolic blood pressure indicators may be attributed to the changes in sodium intake throughout the study period. This is a valuable result of this study. Law et al. showed that a sodium intake decrease of approximately 3,000 mg per day can help decrease the systolic blood pressure indicator by approximately 5–7 mmHg after 5 weeks of intervention (24). The finding in our study is more modest compared to Law et al.’s results; however, in comparison, He FJ et al. (25) reported that when sodium intake decreased by approximately 4,000 per day, the systolic blood pressure indicator decreased by approximately 2 mmHg in people with normal blood pressure, which is similar to our result.

### 4.4 Stress marker

The stress marker CgA showed an increasing trend in the Low free L-Gl group, but the difference was not statistically significant between the two groups. However, the Low free L-Gl group showed an increasing CgA trend between W3 and W6, while the Normal free L-Gl group did not show any changes as reported by Iwamoto et al. (11). They found that the percentage of change in the stress marker from the baseline showed that the group without the umami substance (free L-glutamate) was significantly higher than the group with the umami substance after receiving a sodium-reduced diet for 6 or more days, indicating stress is alleviated when subjects receive free L-glutamate seasonings. These findings could suggest that subjects can adapt more easily to a sodium-reduced diet when they are allowed to consume free L-glutamate-rich seasonings. The reason could be that the free L-glutamate in foods in a reduced-sodium diet may have strengthened the salty-like sensation in the brain (18). The effect of soy sauce odor in enhancing saltiness has also been reported for a salt solution (sodium chloride) with soy sauce odor (26) and in the retro nasal odor of unheated and heated soy sauce (27). However, this study found a better acceptance in the group with a wider variety of seasonings to flavor food.

### 4.5 Limitations in the study

Even though the cross-over study was conducted for 6 weeks, it has some limitations. First, we did not conduct the study in different seasons, such as spring or summer, which could change the diet. Second, we conducted the study only in Hanoi, which may not be representative of all of Vietnam because the seasonings used could be different in different regions. Third, although we collected food records, we focused on seasonings when confirming the 24 h recall with participants. As a result, it was not possible to calculate sodium or potassium intake from these food recalls. Therefore, a larger study covering different areas and seasons with a 24 h food dietary recall is needed. However, to the best of our knowledge, this is the first cross-over study conducted on free-living Vietnamese participants. This study was able to demonstrate that giving educational guidelines to reduce sodium intake but allowing the use of any free L-glutamate-rich seasonings enabled subjects to maintaining a sodium-reduced diet for 6 weeks.

## 5 Conclusion

From these results, we conclude that providing guidelines with educational information about hypertension and the importance of reducing sodium without limiting the variety of free L-glutamate-rich seasonings could improve subjects’ adherence to a sodium-restricted diet more effectively than when the variety of seasonings was limited. These findings may contribute to the development of strategies to achieve sodium reduction goals by controlling the amount of seasonings rich in umami substances rather than avoiding them completely.

## Data availability statement

The raw data supporting the conclusions of this article will be made available by the authors, without undue reservation.

## Ethics statement

The studies involving humans were approved by Ethical Committee of National Institute of Nutrition, Vietnam. The studies were conducted in accordance with the local legislation and institutional requirements. The participants provided their written informed consent to participate in this study.

## Author contributions

VH: Conceptualization, Investigation, Methodology, Project administration, Resources, Supervision, Visualization, Writing – original draft. LT: Conceptualization, Methodology, Software, Formal analysis, Writing – original draft. AW: Methodology, Writing – original draft. SS: Writing – original draft. LH: Investigation, Software, Writing – original draft. NA: Investigation, Writing – original draft. NN: Investigation, Writing – original draft. TI: Formal analysis, Writing – original draft. HM: Methodology, Writing – original draft. HU: Methodology, Writing – original draft. NS: Validation, Writing – original draft. NL: Software, Visualization, Writing – original draft. SY: Conceptualization, Supervision, Validation, Methodology, Writing – original draft.
